# Pain relief after CT-guided pars injections in lumbar spondylolysis: analysis of MRI findings and CT-contrast distribution

**DOI:** 10.1007/s00330-025-11903-8

**Published:** 2025-07-30

**Authors:** Georg Wilhelm Kajdi, Sophia Samira Goller, Christoph Germann, Christoph Johannes Laux, Reto Sutter

**Affiliations:** 1 Department of Radiology, Balgrist University Hospital, Faculty of Medicine, University of Zurich, Zurich, Switzerland; 2 Department of Orthopedics, Balgrist University Hospital, Faculty of Medicine, University of Zurich, Zurich, Switzerland

**Keywords:** MRI, CT, Pain assessment, Spinal injections, Spondylolysis

## Abstract

**Objectives:**

To analyze pain relief in spondylolysis patients with chronic lower back pain (CLBP) after CT-guided bilateral pars injections and investigate MRI findings and CT-contrast distribution as predictors of successful pain relief.

**Materials and methods:**

Patients with bilateral spondylolysis and CLBP receiving CT-guided pars injections were assessed for pain relief 15 min and 1 month post-injection, using a numeric rating scale (NRS) and percentage pain reduction (PPR). Two radiologists assessed lumbar findings on prior MRI and CT-contrast distribution during injection. Successful pain relief was defined as PPR ≥ 50%. Logistic regression was used to investigate imaging predictors of successful pain relief.

**Results:**

In 134 patients (mean age 43.9 ± 16.2 years), average NRS pain score dropped from 5.7 at baseline to 3.7 (PPR 34 ± 47.3%) 15 min post-injection, and to 3.2 (PPR 48 ± 43%) 1-month post-injection (all *p* < 0.001). At 15 min, 56/134 patients (42%) and at 1-month post-injection, 73/134 patients (55%) reported PPR ≥ 50%. Isthmic bone marrow edema (BME) was the only MRI predictor associated with successful pain relief (all *p* ≤ 0.006). Patients with isthmic BME were 6–9 times more likely to show successful pain relief 15 min post-injection, and 2–3 times more likely to show successful pain relief 1 month post-injection (all *p* ≤ 0.046). CT-contrast distribution did not correlate with pain relief (all *p* ≥ 0.27).

**Conclusion:**

Pars injections allowed successful pain relief in 55% of spondylolysis patients after 1 month, with a PPR of 48% on average from baseline. Isthmic BME was an important MRI predictor of successful pain relief for pars injections, whereas CT-contrast distribution was not.

**Key Points:**

***Question**** Imaging predictors of successful pain relief in lumbar spondylolysis patients with CLBP receiving bilateral pars injections are unknown*.

***Findings**** Isthmic BME on MRI was a significant predictor of successful pain relief immediately and 1-month post-injection. CT-contrast distribution did not influence pain relief*.

***Clinical relevance**** CT-guided pars injections offer successful pain relief in spondylolysis patients with CLBP. Isthmic BME on MRI is a significant predictor for successful pain relief, whereas CT-contrast distribution during injection is not*.

**Graphical Abstract:**

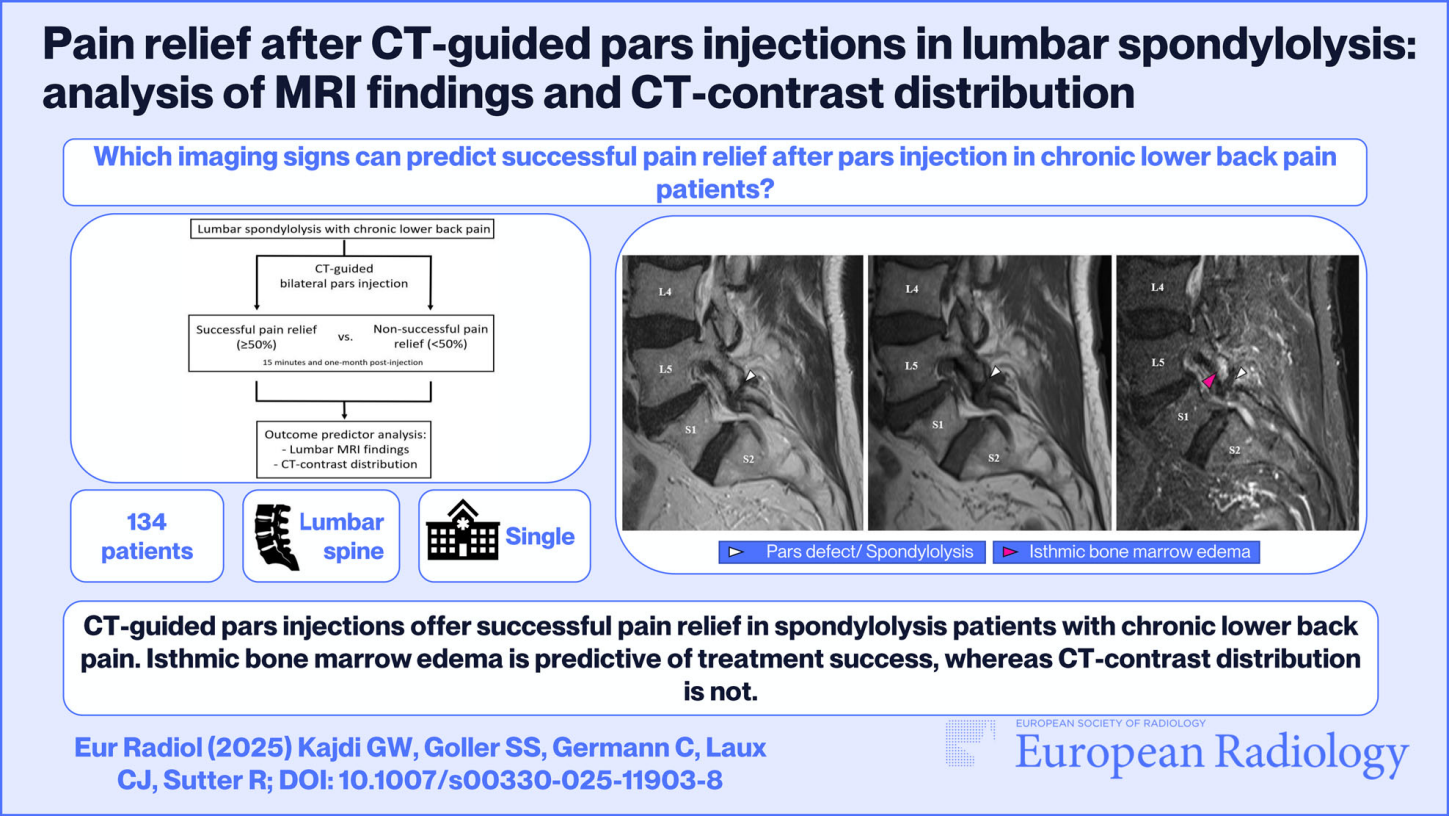

## Introduction

Spondylolysis is characterized by an osseous defect of the pars interarticularis of the vertebral arch. It affects 6% of the adult population and is a common cause of lower back pain [1, 2]. Over 90% of patients with bilateral spondylolysis above 60 years of age also have an associated spondylolisthesis [3, 4].

Imaging plays a pivotal role in its diagnosis, with plain radiographs often serving as the initial modality [5], and more advanced techniques such as CT [6] and MRI offering higher sensitivity in detecting pars defects, especially in early or stress-related lesions [7]. Differentiating spondylolysis from other causes of lower back pain, such as disc herniations, facet joint arthritis, or muscular strains by imaging, is crucial for appropriate patient management [8].

Treatment of symptomatic chronic pars defects includes physiotherapy [9], fluoroscopy [10], and CT-guided [11] pars injections, as well as lumbar fusion as a last resort for refractory cases [2]. Chronic pars defects in refractory lower back pain patients are often found with concomitant lumbar degeneration at the same level or in adjacent spinal levels [12], impeding the imaging identification of the main pain origin. CT-guided pars injections are part of the diagnostic work-up of symptomatic spondylolysis patients: They can have a good therapeutic effect and can also help to identify candidates that are amenable to successful lumbar fusion surgery [13]. Furthermore, for chronic low back pain patients not favoring surgery, repeated pars injections in conjunction with physiotherapy are a valid treatment option [14].

In contrast to more frequently applied spine injections, such as epidural, facet-joint, and nerve root injections, CT-guided pars injections are not well studied. Little is known about their effect on postinterventional pain reduction in spondylolysis patients with lower back pain [11]. As of now, no study has correlated MRI findings or CT-contrast agent distribution during the injection to pain reduction after pars injection. The latter is of special interest, since nociceptive innervation has been demonstrated within pars defects and in their surrounding soft tissues [15, 16], potentially causing different extents of pain reduction, depending on contrast distribution patterns.

The purpose of this study was therefore to evaluate pain reduction after combined local anesthetic and corticosteroid pars injections in patients with bilateral spondylolysis of the lumbar spine, and to assess potential outcome predictors based on lumbar spine MRI findings and CT-contrast distribution during the injection.

## Materials and methods

### Patient selection

This retrospective single-center study was carried out at the radiology department of Balgrist University Hospital in Zurich, and approved by the institutional review board. The study was conducted according to the principles of the Declaration of Helsinki and national ethical standards. All patients included in the study have given written informed consent that allows their health-related data to be used for research purposes.

The institutional picture archiving and communication system (PACS) and clinical information system (CIS) were reviewed for uni-segmental, bilateral spondylolysis patients ≥ 18 years of age, with chronic lower back pain (CLBP, ≥ 3 months) refractory to physiotherapy alone, who had received CT-guided lumbar pars infiltrations between July 2015 and July 2024.

Exclusion criteria included (a) lack of pain assessment, (b) missing pre-infiltration MRI or MRI > 6 months prior to pars injection, uni-lateral pars injection and/or concomitant contralateral facet joint infiltration or nerve root block, (d) incomplete MRI protocols or insufficient MR imaging quality, and (e) lumbar spine surgery before pars injection, and pars injection without iodine-based contrast agent due to possible prior allergic reaction.

### Magnetic resonance imaging

Lumbar spine MRI was conducted on either a 1.5-T unit (Magnetom Avanto Fit and Magnetom Sola, Siemens Healthineers) or a 3-T unit (Magnetom Vida and Magnetom Skyra Fit, Siemens Healthineers) using a 32-channel phased-array spine coil. All patients underwent routine lumbar spine imaging protocols in supine and feet-first positions. All examinations were performed as non-contrast MRI. The sequence parameters varied depending on the scanner (1.5 T vs 3 T) (Supplementary Table 1).

### CT-guided lumbar pars injection

All lumbar pars injections were performed by board-certified, fellowship-trained musculoskeletal radiologists with 5–19 years of experience. The pars injection protocol was standardized to ensure consistency of the intervention. The injections were guided by a 64- or 128-detector row CT (Philips Brilliance, Philips Medical Systems; SOMATOM Definition AS, SOMATOM Definition AS+, or SOMATOM Edge Plus, Siemens Healthineers). Patients had to lie still in a prone position during the procedure. CT imaging was acquired over 1–2 lumbar spine segments according to the previously obtained lateral scout view and confirmed the presence of a bilateral, uni-segmental spondylolysis in all patients. The best access approach for the needle (Sterican, 20 G 7 cm or 21 G, 12 cm, BRAUN) to the pars defect was chosen by the radiologist. Following skin disinfection and subcutaneous application of a local anesthetic, the needle was introduced under CT guidance from posterior until its tip touched the bone at the posterior aspect of the pars defect (Supplementary Fig. 1). For each pars defect, an injection of 0.5 mL iopamidol (Iopamiro 300, 300 mg of iodine per milliliter; Bracco) was performed to verify correct needle tip positioning. Following contrast agent injection, 2 mg (0.5 mL) of the crystalline corticosteroid triamcinolone (Triamcort Depot; Zentiva) was slowly injected on each side. Then 1 mL of 0.2% ropivacaine (Naropin; Astra-Zeneca) was slowly injected into each pars defect.

### Pain evaluation

Pain levels were assessed right before the pars injection (baseline), 15 min after, and at follow-up 1 month (4–5 weeks) post-procedure (either in clinical consultation or by phone consultation). For assessment, the numerical rating scale (NRS) was used, where 0 = no pain, and 10 = worst pain imaginable. Percent change in NRS was calculated from pre-procedure to 15 min and to the first time follow-up post-procedure. The percentage of pain reduction was used to evaluate the primary categorical outcome of successful pain relief for both time points. Based on previous literature, the threshold for success was set at ≥ 50% pain reduction prior to data analysis [11, 17, 18]. All other responses were considered as not improved for our analysis.

### Image analysis

Image analysis was performed using a commercially available PACS workstation (Merlin, Phoenix-PACS). All CT and MRI studies were anonymized and independently reviewed by two board-certified and musculoskeletal fellowship-trained radiologists from the same institution (G.W.K. and S.S.G., with 7 years and 6 years of experience, respectively). No further study-specific pre-readout training was undertaken by any of the readers. Examinations were analyzed in random order, whereby both readers were blinded to clinical data.

**Fig. 1:**
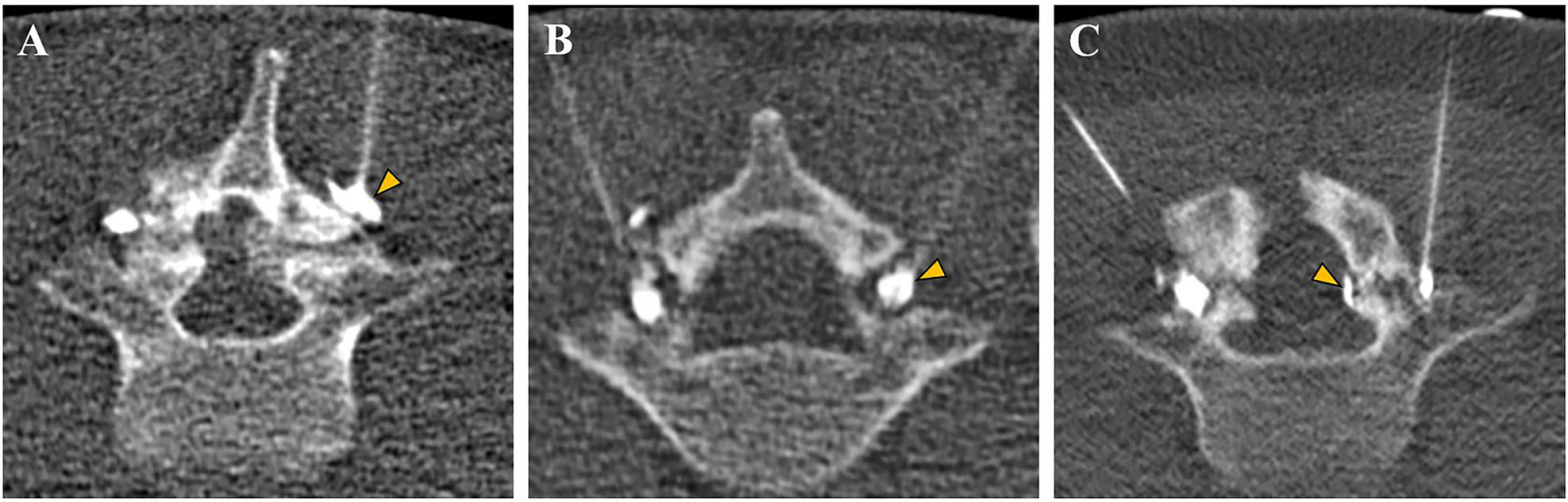
Three different contrast distribution patterns during CT-guided lumbar pars injections in three different patients. Contrast distribution within the soft tissues immediately surrounding the pars defect, coined peri-defect (yellow arrowhead), contrast distribution (**A**). Contrast distribution within the pars defect, coined intra-defect (yellow arrowhead), contrast distribution (**B**). If the contrast agent reached through the pars defect into the epidural space (yellow arrowhead), the pattern was coined trans-defect contrast distribution (**C**). In case different patterns were observed on each side, the deeper contrast distribution pattern was chosen for case classification. Thus, image (**C**) represents a case of trans-defect contrast distribution

For each CT scan, readers had to indicate the pattern of contrast distribution during pars injection as either immediately around the pars defect (peri-defect), within the defect (intra-defect) or with contrast distribution through the defect reaching the epidural space (trans-defect) (Fig. 1). As the pars defect injection was bilateral, it was always the deeper reaching contrast distribution pattern that was chosen for classification.

**Fig. 2:**
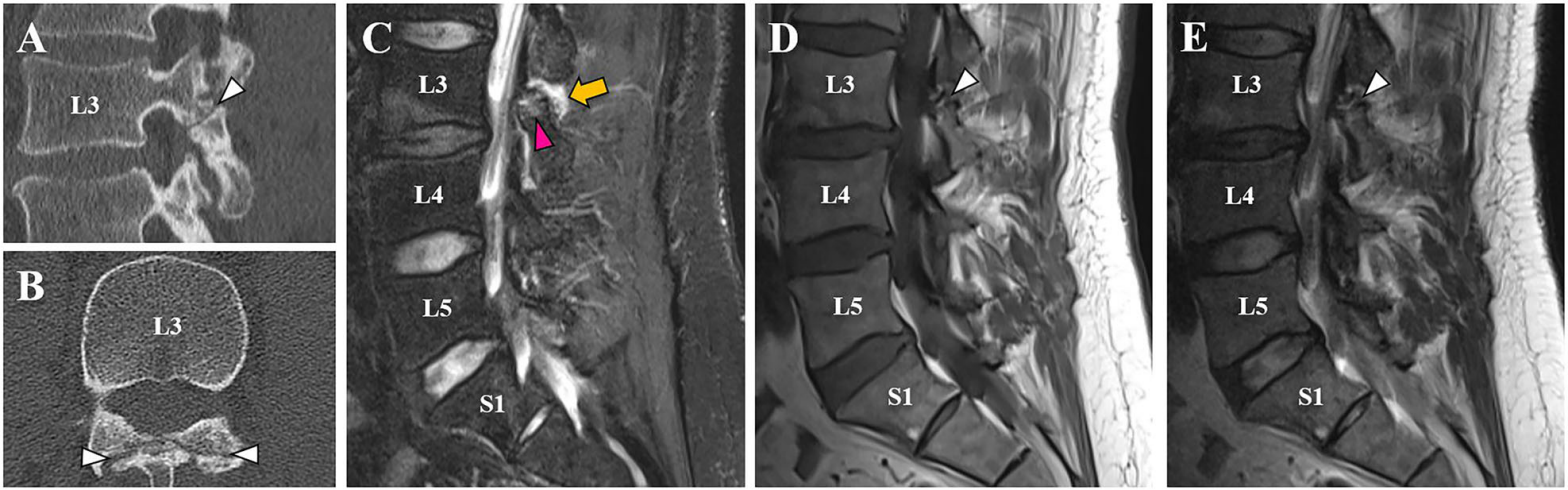
Forty-one-year-old male spondylolysis patient with CLBP, with 33% pain reduction at 15 min, and 100% pain reduction at 1-month follow-up after CT-guided bilateral pars injection. The sagittal (**A**) and axial (**B**) CT images show chronic bilateral spondylolysis at the L3 level with typical sclerotic margins of the lysis borders, which are also identified on the T2w TSE and T1w TSE images as hypointense bands along the pars defect (white arrowheads, **A**–**E**). Sagittal STIR images (**E**) reveal minimal isthmic BME (pink arrowhead) and adjacent soft tissue edema around the spondylolysis (yellow arrow). CLBP, chronic lower back pain; TSE, turbo spin echo

On MRI, readers assessed concomitant signs of degeneration of the lumbar spine, potentially related to lower back pain [19]: The presence of Modic Typ I changes [20] (yes or no), isthmic bone marrow edema (BME) (Supplementary Fig. 2) around the pars defect (yes or no) [21–23] and soft tissue edema [24] and/or synovial cysts [25, 26] in the surrounding soft tissue (yes or no) around the pars defect (Fig. 2 and Supplementary Fig. 3) were included in our analysis. Disc degeneration was graded according to Pfirrmann et al [27], facet joint degeneration according to Weishaupt et al [28], lateral recess stenosis according to Bartynski et al [29], and lumbar foraminal stenosis according to Lee et al [30]. Spondylolisthesis was measured in millimeters, as well as according to Meyerding [31]. Readers evaluated whether degeneration was limited to the segment of the pars defect or affected adjacent lumbar segments (predominantly uni-segmental vs multi-segmental). To qualify for classification as predominantly uni-segmental lumbar degeneration, bone edema, disc degeneration > Pfirrmann Grade 3, and facet joint degeneration > Weishaupt Grade 1 in more than one lumbar segment had to be excluded.

### Statistical analysis

All statistical analyses were performed in SPSS Statistics (v. 29, IBM Corporation).

Normal and non-normal distribution of data was assessed graphically and analytically using Quantile–Quantile plots and the Shapiro–Wilk test. In addition to descriptive statistics, the Wilcoxon signed-rank test was used to assess significant differences in NRS at the different follow-up time points. To ensure consistency and increase methodological transparency, univariate and binary logistic regression analysis of the assessed imaging findings was performed for each reader individually, aligning with established practices [32], as follows: The Mann–Whitney *U*-test and the Chi-square-test were performed to assess significant imaging differences individually between successful pain relief patients and non-successful pain relief patients at the immediate and at the 1-month follow-up. For a more comprehensive model, binary logistic regression analysis was executed to evaluate significant imaging predictors for successful pain relief at both the 15 min and the 1-month follow-up. Considering their non-normal distribution, Spearman’s correlation coefficient (*r*_s_) was used to assess the correlation between NRS pain scores at the 15 min and 1-month follow-up. Inter-reader agreement (IRA) was analyzed by kappa statistics (Cohen’s κ) for all categorical variables. Intraclass correlation coefficient (ICC) was used for continuous variables. For the ICC, a two-way mixed-effects model with absolute agreement definition was applied. Single measures were used, as the raters‘ scores were not averaged in subsequent analysis. The level of agreement for Cohen’s κ was categorized as follows [33]: 0.0 = poor, 0.01–0.20 slight, 0.21–0.40 = fair, 0.41–0.60 = moderate, 0.61–0.80 = substantial, 0.81–1.00 = almost perfect agreement. The level of agreement for the ICC was categorized as follows [34]: 0–0.49 = poor, 0.5–0.74 = moderate, 0.75–0.89 = good, 0.9–1.0 = excellent agreement. All statistical tests were performed two-sided, and a level of significance (α) of 0.05 was used.

## Results

### Patients

From a total of 289 potentially eligible patients who had received CT-guided lumbar pars infiltrations between July 2015 and July 2024, a total of 155 patients had to be excluded during the selection process. This resulted in 134 patients (mean age 43.9 ± 16.2 years, range 18-86 years, 54 females) included in the study (Supplementary Fig. 4), who received lumbar spine MRI either on a 1.5-T unit (*n* = 58 patients, 54.3%), or a 3-T unit (*n* = 76 patients, 56.3%). Detailed demographic data is given in Table 1. Patient age, sex, and body mass index (BMI) did not show significant correlation to percentage pain relief (PPR) and were not significantly associated with successful pain relief at any given time point (all *p* ≥ 0.34).

**Table 1:** Patient demographics, injection levels, pain scores, and pain improvement over time (*n* = 134)

	Mean (SD) or *n* (%)
Age (in years)	43.9 ± 16.2
Male (in %)	59.7%
BMI (in kg/cm^2^)	26.1 ± 4.1
Injection level	
L3	2 (1.5%)
L4	14 (10.4%)
L5	118 (88.1%)
Pain (NRS, 0–10)	
Before injection	5.7 ± 2.2
15 min after injection	3.7 ± 2.5
4 weeks after the injection	3.2 ± 3.0
Pain % change	
15 min after injection	−34.4 ± 47.3
1 month after the injection	−47.5 ± 42.9

*NRS* numeric rating scale, *SD* standard deviation

### Pain response

Average pain assessment on the NRS (0–10) was 5.7 ± 2.2 at pre-injection baseline, 3.7 ± 2.5 immediately (15 min) post-injection, and 3.2 ± 3.0 at the 1-month (4–5 weeks) follow-up. Average PPR from baseline NRS was 34.4% ± 47.3% immediately post-injection (*p* < 0.001) and 47.5% ± 42.9% at the 1-month follow-up (*p* < 0.001) (Table 1). There was a significant (*p* ≤ 0.001), moderate (*r*_s_ = 0.61; 95% confidence interval (CI): 0.49–0.71) correlation between the immediate and 1-month NRS pain response.

**Fig. 3:**
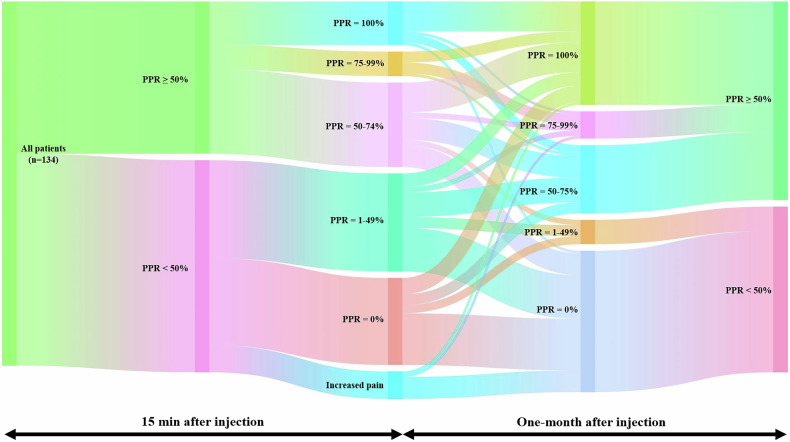
Sankey diagram illustrating percentage pain reduction (PPR) following pars interarticularis injection in CLBP patients with bilateral lumbar spondylolysis (*n* = 134). The diagram maps patient response over two time points: 15 min and 1 month post-injection. The second and last columns of nodes categorize patients broadly into successful (PPR ≥ 50%) and non-successful (PPR < 50%) treatment response, while the node columns in between provide more detailed subdivisions (increased pain, PPR = 0%, 1–49%, 50–74%, 75–99%, and 100%). The vertical width of each node and of its connecting flows is proportional to the fraction of the total study population. The connecting flows indicate a transition in individual pain response over time

Fifty-six patients (41.8%) reported successful pain relief (≥ 50%) 15 min post-injection and 73 patients (54.5%) reported successful pain relief at the 1-month follow-up, showcasing a significant (*p* = 0.009) increase in the proportion of successful treatment response from immediate to 1-month follow-up (Table 2 and Fig. 3). A subset of patients showed complete pain relief (PPR = 100%) at the 15 min (*n* = 16, 12%) and at the 1-month (*n* = 38, 28.3%) follow-up.

**Table 2:** Proportion of responders and non-responders at the 15 min and 1-month follow-up post-injection (*n* = 134)

Pain relief	Immediate response (15 min post-injection)		1-month response (follow-up)	
	*n*	%	*n*	%
Responders	56	41.8	73	54.5
≥ 50% to < 75%	31	23.1	25	18.7
75% to < 100%	9	6.7	10	7.5
100%	16	12	38	28.3
Non-responders	78	58.2	61	45.5
> 0% to < 50%	36	26.9	9	6.7
0%	32	23.9	52	38.8
Increased pain	10	7.4	0	0

### Imaging findings

In Supplementary Table 2, the absolute number and percentage of patients with all assessed MR imaging findings are summarized. Of the assessed imaging findings uni-segmental lumbar degeneration (reader 1: *p* = 0.047; reader 2: *p* = 0.044), presence of isthmic BME around the pars defect (reader 1 and 2: *p* < 0.001) and presence of surrounding soft tissue edema and/or synovial cysts (reader 1: *p* = 0.013; reader 2: *p* = 0.046) were individually significantly associated with successful pain relief 15 min post-injection. Presence of isthmic BME around the pars defect (reader 1: *p* = 0.006; reader 2: *p* = 0.005) was the only individual imaging finding that was also significantly associated with successful pain relief at the 1-month follow-up (reader 1 and 2: *p* = 0.005). All other imaging findings (CT contrast distribution, uni- vs multi-segmental degeneration, disc degeneration, Modic type I changes, facet joint degeneration, spondylolisthesis in mm, spondylolisthesis according to Meyerding, foraminal stenosis, lateral recess stenosis, and soft tissue edema and/or synovial cysts) did not show statistical significance (all *p* ≥ 0.07) at the 1-month follow-up. The prevalence of each imaging finding based on non-successful vs successful treatment response is detailed in Supplementary Table 3.

Binary logistic regression analysis was performed for each of the two readers individually using the 11 imaging findings compared to successful pain relief (yes or no), for 15 min and for 1 month post-injection. The four resulting prediction models for treatment success, including their respective accountability of variability, overall accuracy, sensitivity, specificity, positive (PPV), and negative predictive value (NPV), are summarized in Table 3.

**Table 3:** Prediction models for successful pain relief at 15 min and 1-month post-injection by the reader based on the presence of all 11 assessed imaging findings

Model	Timepoint	Reader	Explained variability	Accuracy	Sensitivity	Specificity	PPV	NPV
1	15 min	1	43.3%	72.0%	64.3%	76.9%	66.7%	75.0%
2	15 min	2	40.1%	73.0%	62.5%	80.8%	70.0%	75.0%
3	1 month	1	27.3%	68.0%	72.6%	62.3%	69.6%	65.5%
4	1 month	2	23.0%	66.4%	71.2%	60.7%	68.4%	63.8%

*NPV* negative predictive value, *PPV* positive predictive value

Only the independent variable isthmic BME around lysis made a statistically significant contribution to both models for both readers (all *p* ≤ 0.046) with an odds ratio (OR) of 6.2 (95% CI: 2.0; 18.9) (reader 2: OR: 8.9; 95% CI: 3.0, 26.0) at 15 min post injection and with an OR of 2.8 (95% CI: 1.0; 7.4) (reader 2: OR: 2.6; 95% CI: 1.1, 6.3) at the 1 month follow-up. 64% of patients with isthmic BME experienced successful pain relief at immediate follow up and 70% of them at 1-month follow-up.

### IRA

IRA for all assessed imaging findings between the two readers ranged from moderate (facet joint degeneration, lateral recess stenosis), over substantial (CT contrast distribution, disc degeneration, soft tissue edema and/or synovial cysts), to almost perfect (segment degeneration, endplate edema (Modic I), spondylolisthesis in mm, spondylolisthesis (Meyerding), isthmic BME around lysis) with a detailed summary in Supplementary Tables 2 and 3.

## Discussion

In this study, we demonstrated that CT-guided pars injections are a helpful tool in patients with bilateral spondylolysis and CLBP for diagnosis and therapy, with a significant percentage pain reduction (PPR) of 34% at 15 min, and of 48% 1-month post-injection. Isthmic BME on MRI was a significant predictor for successful pain relief after pars injection. As of today, our study comprises the largest cohort for which immediate and 1-month pain relief after CT-guided lumbar pars injection was evaluated. Furthermore, it is the only study that has compared MR imaging predictors and contrast distribution during the CT-guided injection to successful immediate and 1-month pain relief after pars injection.

Spondylolysis or pars defects are known to have a predilection for the male sex (2.5-to-1 male-to-female ratio) [35] and for the L5 segment, reported to be affected in 85–95% of cases [36]. Our study cohort represented these observations well, with 60% of affected patients being of male sex and 88% having L5 pars defects.

Wald et al (*n* = 59) have previously evaluated pain and disability scores in patients with chronic lumbar pars defects after pars injection [11], with an average baseline NRS score (5.4 ± 2.1) similar to our study (5.7 ± 2.2). At the 1-month follow-up, our study showed slightly lower scores on the NRS pain scale compared to the previous study (3.2 ± 3.0 vs 3.7 ± 2.5) and a higher proportion of patients reporting successful (same threshold: PPR ≥ 50%) pain relief (55% vs 43%). This is especially noteworthy, as the follow-up in our study was set at 4 weeks compared to 2 weeks post-injection in the previous study. Our more favorable outcomes might be due to discrepancies in methodology, such as the use of triamcinolone instead of betamethasone. A study by McCormick et al (*n* = 1021) had shown more frequent successful pain relief at short-term follow-up for epidural triamcinolone injection compared to particulate betamethasone [37]. Thus, the same might be true for pars injections. However, smaller randomized controlled clinical trials by Manchikanti et al [38, 39] found no significant difference in pain relief for different particulate and non-particulate corticosteroids in epidural spine injections within a 2-year follow-up, highlighting the lack of reliable data to prove long-term differences in outcome based on the applied corticosteroid.

Of all the assessed MR imaging findings, only isthmic BME was shown to be a significant predictor for successful pain relief at 15 min and at 1-month post-injection. 70% of patients showing isthmic BME reported successful pain relief, compared to only 55% in the overall study cohort. Patients with isthmic BME were six to nine times more likely to report successful pain relief at the 15 min mark and two to three times more likely to report successful pain relief at 1-month post-injection, making it a reliable and easily reproducible (κ: 0.94, SE: 0.03) predictor for treatment success. This correlation is compatible with observations by Borg et al (*n* = 646), who found isthmic BME to be resolving in association with functional improvement and pain decrease during the natural course of spine degeneration, even though that was only an observational study, and only four of their patients suffered from spondylolysis [40]. Interestingly, isthmic BME is readily acknowledged in the literature on pars defects to be indicative of bone healing potential in acute and subacute stages of spondylosis [21–23, 41, 42]. In contrast to this, our study focused solely on pseudoarthrotic chronic pars defects with no spontaneous healing tendency [2, 43, 44]. Thus, isthmic BME plays a different clinical role in this stage of the disease as it implicates a treatable cause for pain rather than indicating a chance for spontaneous healing.

Despite not reaching significance in the logistic regression analysis, uni-segmental degeneration of the lumbar spine and soft tissue edema and/or synovial cyst around lysis were individually significantly associated with successful immediate pain relief. This is intuitive from a clinician’s perspective, as uni-segmental degeneration increases the chance of the pain origin being located in the same segment. Furthermore, pars defects and their surrounding soft tissues have been shown to have nociceptive innervation [15, 16], thus making soft tissue edema and/or protruding synovial cysts in the same localization a plausible irritant and imaging correlate for pain. Although neither of the two imaging findings was individually correlated to 1-month success in this study, the window between immediate and 1-month pain relief in these patients might give a chance for more rigorous physiotherapy with possible synergistic effects on later pain relief.

The role of CT contrast distribution pattern for clinical outcomes in lumbar spine injections has been studied for nerve root blocks [45]. No studies on the matter exist for lumbar pars injection. Expert opinion suggests aiming for contrast distribution within the pars defect [11]. However, even though the needle was routinely centered within the posterior aspect of the defect, contrast distribution in our study was not readily foreseeable, and was mainly split between peri-defect soft tissue contrast distribution (*n* = 66; 49.3%) and intra-defect contrast distribution (*n* = 55; 41.0%). Similar to the observations by Germann et al for CT-guided lumbar nerve block [45], the contrast distribution pattern did not significantly impact pain response at any time point after pars injection (all *p* ≥ 0.56).

Limitations of this study need to be acknowledged. First, this study is a short-term cohort outcome study and lacks the control group (e.g., injection of saline) of a randomized controlled clinical trial. To reduce the impact of covariates, great care was taken to homogenize the patient cohort by including solely spondylolysis patients with CLBP that was refractory to prior physiotherapy. Continued physiotherapy, and concomitant oral pain medication during the 1-month time interval after pars injection, were not systematically documented, and must be considered a confounder, possibly offering synergistic effects to the pars infiltration in terms of pain relief. Furthermore, the development of bone edema at the 1-month mark was not followed up with imaging. Other than CT-contrast distribution, all assessed imaging findings were solely assessed using lumbar spine MRI. CT might offer a more accurate depiction of discrete degenerative bony changes of the lumbar spine. Finally, physicians should be cautious not to solely base treatment decisions upon the presence of isthmic BME, since its absence does not preclude treatment success. However, chronic CLPB patients with isthmic BME along their pars defects, can be reassured to be more favorable candidates for successful pars injection treatment. Nonetheless, we must admit that the predictability of success in interventional pain therapy remains overall limited, and continued investigation is warranted to further improve the predictability of patient outcomes.

In conclusion, this study reveals that CT-guided injections of chronic lumbar pars defects result in successful pain relief in 55% of patients at 1-month post-injection, with a significant pain reduction of 48% on average from baseline. Isthmic BME on MRI was a significant imaging predictor of successful pain relief after pars injection, whereas CT contrast distribution was not.

## Supplementary information

ELECTRONIC SUPPLEMENTARY MATERIAL

## Abbreviations

BME: Bone marrow edema
BMI: Body mass index
CI: Confidence interval
CLBP: Chronic lower back pain
ICC: Intraclass correlation coefficient
IRA: Inter-reader agreement
NPV: Negative predictive value
NRS: Numeric rating scale
OR: Odds ratio
PACS: Picture archiving and communication system
PPR: Percentage pain relief
PPV: Positive predictive value
