# Supplementary material for: Pain relief after CT-guided pars injections in lumbar spondylolysis: analysis of MRI findings and CT-contrast distribution
Source: Eur Radiol. 2025 Jul 30;36(2):1272–81. doi: 10.1007/s00330-025-11903-8 (PMC12953276; doi:10.1007/s00330-025-11903-8)
Supplement: Supplementary file 1 — ELECTRONIC SUPPLEMENTARY MATERIAL [file 330_2025_11903_MOESM1_ESM.pdf]

Pain relief after CT-guided pars injections in lumbar spondylolysis: Analysis of MRI findings and CT-contrast distribution

ELECTRONIC SUPPLEMENTARY MATERIAL

Supplementary Figures

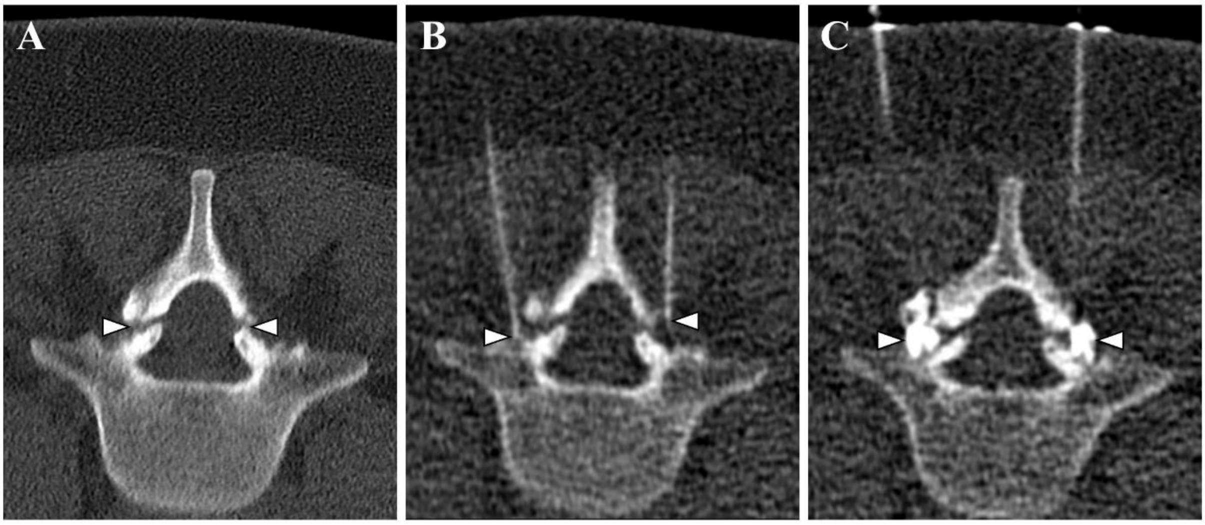

Supplementary Figure 1: CT-guided contrast injection into the pars defect in a 33-year old female patient with bilateral spondylolysis and chronic lower back pain.

For the intervention the patients were positioned in prone and head-first position in the scanner. After visualization of the bilateral pars defect (orange arrowheads) **(A)**, the needles were introduced under CT guidance from posterior or slightly posterolateral and advanced until both needle tips touched the respective bone at the posterior and/ or lateral aspect of the pars defect (orange arrowheads) **(B)**. When needle-to-bone contact was reached, the CT-contrast agent was injected bilaterally (orange arrowheads) **(C)** with three possible contrast distribution patterns (in this case: intra-defect contrast distribution bilaterally). Contrast injection was immediately followed by corticosteroid and local anesthetic injection on both sides.

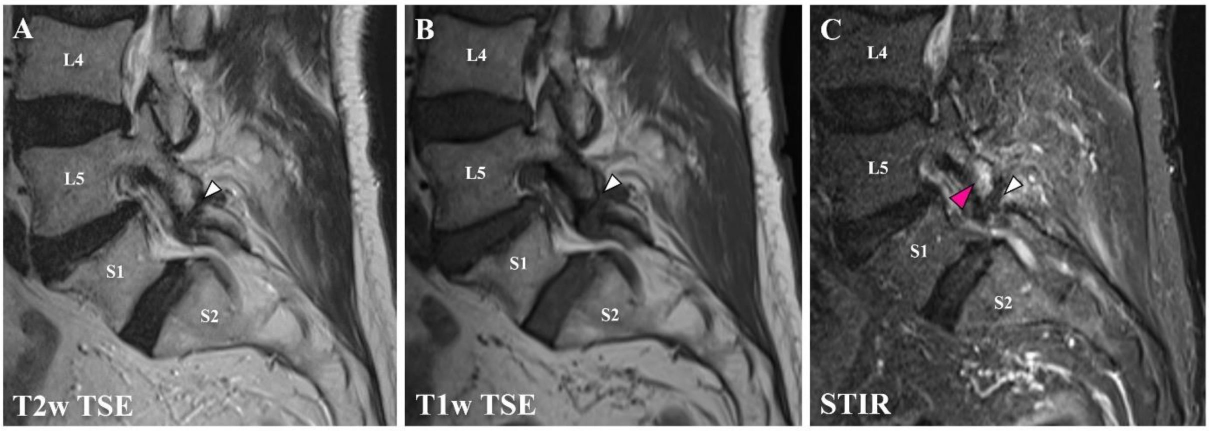

Supplementary Figure 2: Isthmic bone marrow edema in a 74-year old male CLBP patient with bilateral spondylolysis, with successful immediate and one-month pain relief (=70%) following CT-guided bilateral pars injection.

The chronic L5 pars defect (white arrowhead) is visible on T2w **(A)**, T1w **(B)** TSE and STIR **(C)** images. The sagittal STIR **(C)** image illustrates the adjacent isthmic bone marrow edema in L5 (pink arrowhead).

CLBP, chronic lower back pain; TSE, turbo spin echo

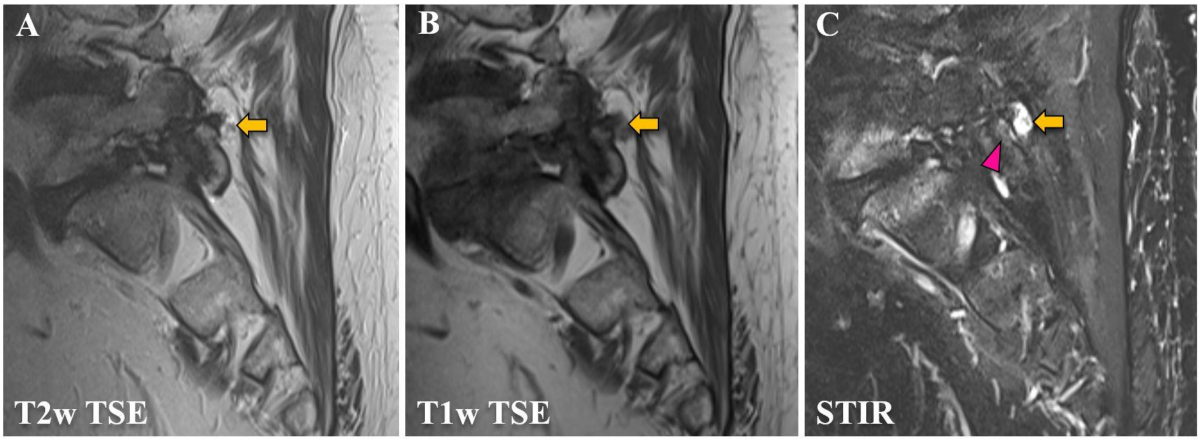

Supplementary Figure 3: MRI findings in a 70-year old male CLBP patient with spondylolysis, with successful immediate and one-month pain relief (=50%) following CT-guided bilateral L5 pars injection.

A synovial cyst is observed (orange arrow) on T2w TSE **(A)**, T1w TSE **(B)** and STIR **(C)** images, with extension posterior to the bony confinements of the pars defect into the paraspinal soft tissues. Minimal isthmic bone marrow edema on sagittal STIR (pink arrowhead, **C**) around the pars defect was observed as well.

CLBP, chronic lower back pain; TSE, turbo spin echo

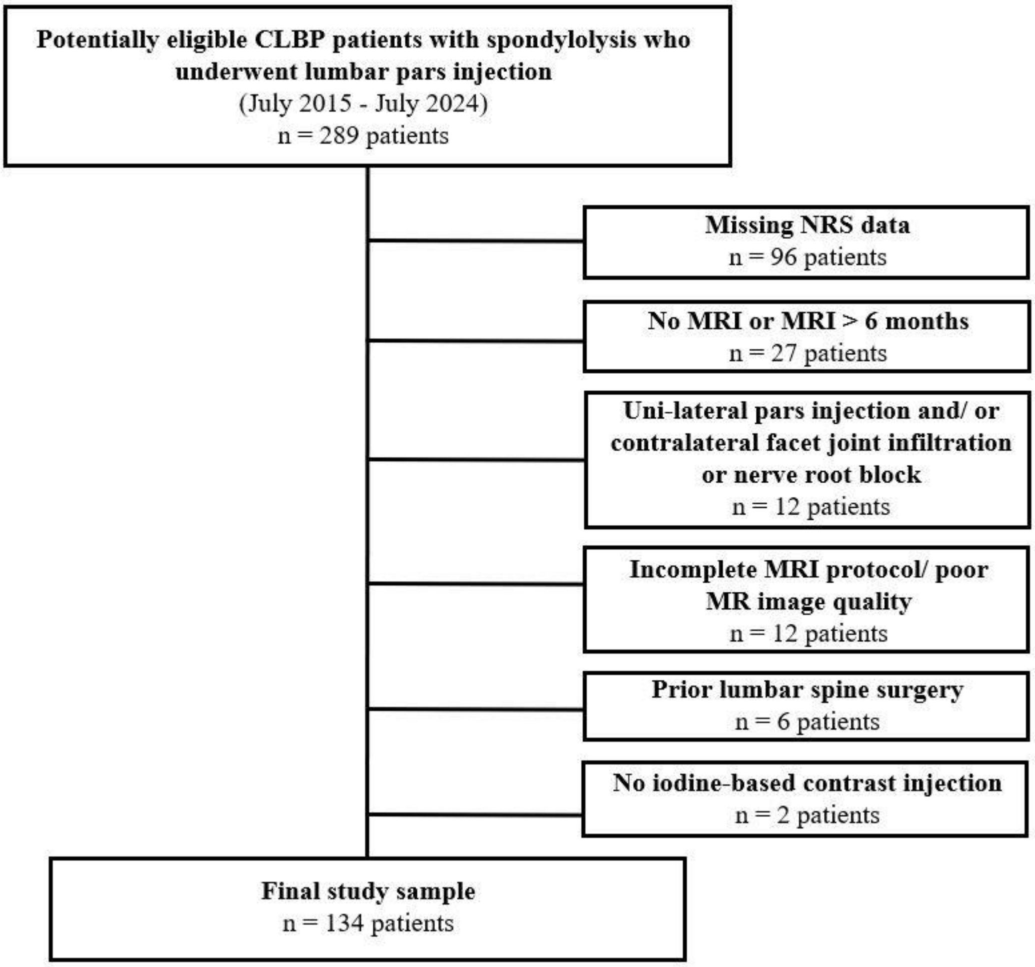

Supplementary Figure 4: Flowchart illustrating the patient selection process.

From n = 289 potentially eligible patients, 155 patients had to be excluded during the selection process, resulting in a final study sample of 134 patients.

CLBP, chronic lower back pain; NRS, numeric rating scale

Supplementary Tables:

Supplementary Table 1: Sequence parameters at 1.5T and 3T.

**1.5T MRI**				
**Sequence**	**T1 TSE sag**	**T2 TSE Sag**	**STIR sag**	**T2 TSE tra**
**TR [ms]**	**504**	**5590**	**4500**	**4200**
**TE [ms]**	**9.5**	**95**	**38**	**87**
**Bandwidth [Hz/px]**	**200**	**200**	**191**	**191**
**Slices [n]**	**28**	**28**	**28**	**22**
**Slice thickness [mm]**	**3**	**3**	**3**	**3**
**Spacing [mm]**	**3.3**	**3.3**	**3.3**	**3.3**
**Matrix**	**352x282**	**352x282**	**320x256**	**384x269**
**FOV [mm]**	**299**	**299**	**300**	**220**
**TA [min:s]**	**1:19**	**1:18**	**1:52**	**1:28**
**3T MRI**				
**Sequence**	**T1 TSE sag**	**T2 TSE sag**	**STIR sag**	**T2 TSE tra**
**TR [ms]**	**524**	**3200**	**5500**	**3500**
**TE [ms]**	**9.6**	**87**	**37**	**87**
**Bandwidth [Hz/px]**	**253**	**252**	**261**	**252**
**Slices [n]**	**28**	**28**	**28**	**25**
**Slice thickness [mm]**	**3**	**3**	**3**	**3**
**Spacing [mm]**	**3.3**	**3.3**	**3.3**	**3.6**
**Matrix**	**304x274**	**336x336**	**304x274**	**432x346**
**FOV [mm]**	**300**	**300**	**300**	**220**
**TA [min:s]**	**1:44**	**2:14**	**1:39**	**2:00**

Hz, Hertz; FOV, field of view; sag, sagittal; STIR, short tau inversion recovery; px, pixel; TA, acquisition time; TE, echo time; TR, repetition time; tra, transversal; TSE, turbo-spin-echo.

Supplementary Table 2: Imaging finding by reader & inter-reader agreement (IRA) (n=134)

Imaging finding, n (%)	Reader 1	Reader 2	κ / ICC* (SE)	IRA	P
CT					
Contrast distribution			0.72 (0.06)	substantial	<0.001
Peri-defect	66 (49.3)	55 (41.0)			
Intra-defect	55 (41.0)	66 (49.3)			
Trans-defect	13 (9.7)	13 (9.7)			
MRI					
Segment degeneration			0.82 (0.05)	almost perfect	<0.001
Predominantly uni-segmental	80 (59.7)	70 (52.2)			
Multi-segmental	54 (40.3)	64 (47.8)			
Disc degeneration			0.72 (0.05)	substantial	<0.001
Grade 0	3 (2.2)	3 (2.2)			
Grade 1	8 (6.0)	8 (6.0)			
Grade 2	12 (9.0)	12 (9.0)			
Grade 3	30 (22.4)	34 (25.4)			
Grade 4	36 (26.9)	38 (28.4)			
Grade 5	45 (33.5)	39 (29.1)			
Modic type I changes			0.89 (0.04)	almost perfect	<0.001
present	77 (57.5)	80 (59.7)			
not present	57 (42.5)	54 (40.3)			
Facet joint degeneration			0.57 (0.06)	moderate	<0.001
Grade 0	2 (1.5)	8 (6.0)			
Grade 1	79 (58.9)	69 (51.5)			
Grade 2	41 (30.6)	50 (37.3)			
Grade 3	12 (9.0)	7 (5.2)			
Spondylolisthesis			0.97 (0.02)	almost perfect	<0.001
Grade 0	21 (15.7)	21 (15.7)			
Grade 1	95 (70.9)	95 (70.9)			
Grade 2	17 (12.7)	17 (12.7)			
Grade 3	1 (0.7)	1 (0.7)			
Spondylolisthesis (in mm)	5.8 ± 4.0	6.2 ± 4.1	0.96 (0.01)	excellent	<0.001
Foraminal stenosis			0.75 (0.05)	substantial	<0.001
Grade 0	32 (23.9)	26 (19.4)			
Grade 1	38 (28.4)	42 (31.3)			
Grade 2	28 (20.9)	33 (24.6)			
Grade 3	36 (26.8)	33 (24.6)			
Lateral recess stenosis			0.52 (0.06)	moderate	<0.001
Grade 0	103 (76.9)	86 (64.2)			
Grade 1	17 (12.7)	33 (24.6)			
Grade 2	8 (6.0)	11 (8.2)			
Grade 3	6 (4.5)	4 (3.0)			
Isthmic BME around lysis			0.94 (0.03)	almost perfect	<0.001
present	50 (37.3)	50 (37.3)			
not present	84 (62.7)	84 (62.7)			
Soft tissue edema/ synovial cyst			0.75 (0.06)	substantial	<0.001
present	92 (68.7)	98 (73.1)			
not present	42 (31.3)	36 (26.9)			

*BME, bone marrow edema, CI, confidence interval; ICC, intraclass correlation coefficient; IRA, inter-reader agreement; SE, standard error; *ICC was only used for spondylolisthesis (in mm)*

Supplementary Table 3: Imaging findings based on non-successful (PPR <50%) vs. successful (PPR ≥50%) treatment response at 15 minutes and one-month post-injection.

	PPR at 15 minutes post-injection			PPR at one-month post-injection		
**Imaging finding, n (%*)**	**<50%**	**≥50%**	**P**	**<50%**	**≥50%**	**P**
**CT**						
**Contrast distribution**			0.50			0.27
Peri-defect	42 (53.8)	24			39	
Intra-defect	26 (33.3)	29			28	
Trans-defect	10 (12.8)	3			6	
**MRI**						
**Segment degeneration**			0.047			0.12
Predominantly uni-segmental	41 (52.6)	39		32	48	
Multi-segmental	37 (47.4)	17		29	25	
**Disc degeneration**			0.56			0.82
Grade 0	1 (1.3)	2		1	2	
Grade 1	3	5		2	6	
Grade 2	7	5		7	5	
Grade 3	17	13		13	17	
Grade 4	25	11		18	18	
Grade 5	25	20		20	25	
**Modic type I changes**			0.95			
present	45	32		30	47	
not present	33	24		31	26	
**Facet joint degeneration**			0.64			0.83
Grade 0	0	2		0	2	
Grade 1	46	33		38	41	
Grade 2	26	15		18	23	
Grade 3	6	6		5	7	
**Spondylolisthesis**			0.83			0.67
Grade 0	11	10		8	13	
Grade 1	57	38		45	50	
Grade 2	10	7		8	9	
Grade 3	0	1		0	1	
**Spondylolisthesis (in mm)**	6.0 ± 3.9	5.8 ± 3.9	0.78	6.2 ± 4.1	5.6 ± 4.0	0.48
**Foraminal stenosis**			0.67			0.62
Grade 0	21	11		12	20	
Grade 1	21	17		19	19	
Grade 2	14	14		14	14	
Grade 3	22	14		16	20	
**Lateral recess stenosis**			0.13			0.14
Grade 0	56	47		43	60	
Grade 1	12	5		11	6	
Grade 2	8	0		4	4	
Grade 3	2	4		3	3	
**Isthmic BME around lysis**			<0.001			0.006
present	18	32		15	35	
not present	60	24		46	38	
**Soft tissue edema/ synovial cyst**			0.013			0.068
present	47	45		37	55	
not present	31	11		24	18	

BME, bone marrow edema; PPR, percentage pain relief; *percentage of patients within each response subgroup
